# Contactless Deformation Monitoring of Bridges with Spatio-Temporal Resolution: Profile Scanning and Microwave Interferometry

**DOI:** 10.3390/s22239562

**Published:** 2022-12-06

**Authors:** Florian Schill, Chris Michel, Andrei Firus

**Affiliations:** 1 Institute of Geodesy—Geodetic Measuring Systems and Sensor Technology, Technical University of Darmstadt, Franziska-Braun-Straße 7, 64287 Darmstadt, Germany; 2 Institute of Photogrammetry and Remote Sensing, Karlsruhe Institute of Technology, Englerstraße 7, 76131 Karlsruhe, Germany; 3 iSEA Tec GmbH, Flughafen 76, 88046 Friedrichshafen, Germany

**Keywords:** structural health monitoring (SHM), dynamic deformation monitoring, short-time, bridges, terrestrial laser scanning (TLS), profile scanning, microwave interferometry (MI)

## Abstract

Against the background of an aging infrastructure, the condition assessment process of existing bridges is becoming an ever more challenging task for structural engineers. Short-term measurements and structural monitoring are valuable tools that can lead to a more accurate assessment of the remaining service life of structures. In this context, contactless sensors have great potential, as a wide range of applications can already be covered with relatively little effort and without having to interrupt traffic. In particular, profile scanning and microwave interferometry, have become increasingly important in the research field of bridge measurement and monitoring in recent years. In contrast to other contactless displacement sensors, both technologies enable a spatially distributed detection of absolute structural displacements. In addition, their high sampling rate enables the detection of the dynamic structural behaviour. This paper analyses the two sensor types in detail and discusses their advantages and disadvantages for the deformation monitoring of bridges. It focuses on a conceptual comparison between the two technologies and then discusses the main challenges related to their application in real-world structures in operation, highlighting the respective limitations of both sensors. The findings are illustrated with measurement results at a railway bridge in operation.

## 1. Introduction

Against the background of an aging infrastructure and because of the clear trend towards the development of faster and heavier vehicles, the condition assessment of existing railway bridges is becoming an increasingly challenging task for civil engineers, especially when important decisions have to be made about costly replacement or rehabilitation measures. In this context, the accurate knowledge of the real structural behavior is a valuable tool in the condition assessment process, which in many cases can lead to a significant extension of the remaining service life and thus to considerable benefits for both the bridge owners and society.

The actual structural behaviour is usually assessed by experimental investigations, which can include measurements of accelerations, velocities, strains, slopes or temperatures [1,2,3]. Furthermore, displacement measurements based on linear variable displacement transducers (LVDT) are used for assessing the relative displacement at the supports [1], between adjacent superstructures of the same bridge [4] or for monitoring the width of existing cracks. These types of displacement measurements are basically possible, since a fixed reference point can be used for installing the sensor.

Another important parameter of the structural behavior would be the absolute vertical displacement of the bridge deck. This could provide direct information about the actual stiffness of the structure, which in turn could be incorporated into the updating process of the structural model (e.g., finite element models) [5,6]. However, the direct measurement of absolute displacements using classical LVDTs is usually very complex or even impossible due to the lack of fixed reference points [3]. In order to fill this gap, remarkable advances in the field of contactless displacement measurement methods have been made in recent years [7,8]. They allow the measurement of structural displacements without the need for installing any sensors on the structure. This represents a great advantage, especially in relation to the investigation of bridges in operation, which often require temporary closures for physical access to the structure.

Applicable technologies for contactless displacement measurements are, e.g., laser vibrometry [9,10], image-assisted total station (IATS) [11], terrestrial laser scanning (TLS) [7] and microwave interferometry (MI) [12,13]. Compared to other contactless measurement technologies that allow measurements at only one discrete point, microwave interferometry and TLS additionally enable spatially distributed acquisition of the structural response. The spatial resolution of those sensors offers the advantage that larger areas of the structure can be monitored with only one sensor, allowing a deeper understanding of the structural response in an efficient way. Therefore, these two sensors are the focus of this paper. Typically, TLS are used in 3D mode to capture the static environment. In addition, some models can also be used in profile mode (2D) and are hereafter referred to as profile scanners. For the dynamic measurement of bridge structures, only the second mode is relevant [7], as it requires a high temporal resolution.

For a better understanding of the investigations presented in this paper and their classification in the field of bridge monitoring, the investigated scenario for the contactless monitoring of bridges with spatio-temporal resolution is defined first:

- Measurements without any additional elements on the object (no reflectors)
- Requirement of temporal and spatial resolution
- Parameter of interest: vertical displacements
- Resulting uncertainties in the sub-millimetre range
- Measurement range < 100 m;
- Measurement duration < 10 min.

In such an application scenario, only two of the above mentioned technologies remain: microwave interferometry and profile scanning. In the first part of this paper, a conceptual comparison of the two measurement systems based on this scenario and the sensor specifications will highlight unique limitations for the identified application scenario. Subsequently, it will be shown how the identified problems can be illustrated with the results of experimental investigations on a large multi-span truss railway bridge (height ≥10 m, span lengths ≥80 m). These investigations were in the framework of a condition assessment process and could only be realized with reasonable effort by contactless displacement measurements. The measurement concept used in this investigation is based on the application scenario described above and includes the recording of absolute dynamic displacements in a vertical direction at various points along the structure induced by train passages.

The simultaneous use of both sensors enables a systematic and comprehensive comparison with regard to their applicability and the uncertainties occurring in measurements on bridge structures. Moreover, the extension of the investigation to a railway bridge in operation provides indications of further limitations in the application that clearly go beyond the results of the conceptual comparison. For example, systematic errors of microwave interferometry with respect to the derivation of absolute displacement values, which has already been discussed in the literature [8], cannot only be quantified but also corrected by using both systems simultaneously.

## 2. Investigated Sensors

In the following section, the investigated sensors are presented briefly. First, however, an overview of relevant work in the field of contactless monitoring of bridges with microwave interferometry and profile scanning in general is presented.

### 2.1. Related Work

Schill [7] describes in great detail the basics of profile scanning and also provides extensive application examples for a variety of typical use cases, such as bridges, wind turbines, etc. Furthermore, advanced processing algorithms are presented that enable the universal use of a profile scanner. Further publications on the subject of profile scanning also deal with bridges [14,15,16,17], noise barriers [18] and wind power plants [14,19,20,21,22,23]. In these publications, the sensor performance is regularly evaluated through comparisons with conventional sensors, such as LVDTs or accelerometers, proving that a profile scanner can be used reliably for a wide range of structures and applications.

For the application of microwave interferometry, good overviews are given by Gentile and Bernardini [12], Bernardini et al. [13], Rödelsperger et al. [24]. Microwave interferometry has also been applied to various types of structures in recent years: bridges [8,25,26,27,28,29,30,31], wind power plants [21], telecommunication towers [32,33], chimneys [24] or urban buildings [34,35,36]. Publications dealing with the performance evaluation through comparisons with conventional sensors are, for example, [8,35,37,38]. In summary, microwave interferometry shows excellent performance in determining the natural frequencies of the structures under investigation, while the derivation of absolute displacements often shows large systematic errors even though additional reflectors were attached to the structures in almost all studies. The latter fact is rather problematic, especially considering that the manufacturers of microwave interferometers specify an uncertainty in the low sub-millimetre range [39].

### 2.2. IDS IBIS-S

**Figure 1:**
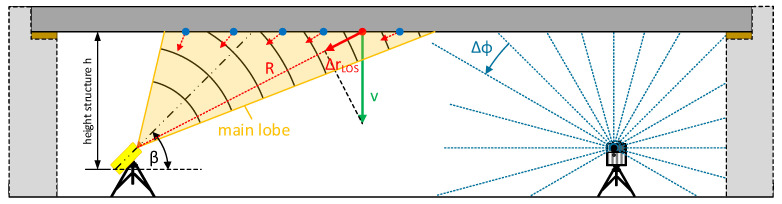
Schematic illustration showing the application of microwave interferometry and profile scanning for the deformation monitoring of bridges.

The microwave interferometry system IBIS-S [39] enables the detection of 1D line-of-sight (LOS) displacements $\Delta r_{LOS}$ based on amplitude and phase measurements. A corresponding schematic representation can be seen in Figure 1. For this purpose, the IBIS-S emits electromagnetic waves in the microwave spectrum (K_u_ band, 17.4 mm wavelength). Due to a stable phase reference of successive measurements, it is possible to evaluate not only the amplitude (intensity) but also the phase in particular. Specifically from the phase, the relative movement of objects in the sensor’s line of sight can be derived by means of interferometry, i.e., the difference of the phase of two measurements, also known as the interferometric phase ϕ.

By modulating the frequency of the emitted signal, multiple objects can be differentiated by their LOS range R to the sensor. The used modulation bandwidth of 200 MHz results in a minimum required distance between objects of 0.75 m to detect them separately.

The relationship between the line-of-sight displacement $\Delta r_{LOS}$ and the interferometric phase ϕ is given by:

(1) $$\phi =-\frac{\lambda }{4\pi }\Delta r_{LOS}$$

The interferometric phase is always in the range $\pm \frac{\lambda }{4}$. Thus, movements that exceed a quarter of the wavelength between two measurements cannot be detected as such, since no absolute range is determined. For more information concerning the function of microwave interferometers see [30,40]. Further details on the projection of the LOS displacements $\Delta r_{LOS}$ using the range R and the height h are discussed in Section 3.5.

### 2.3. Zoller+Fröhlich IMAGER 5016

Terrestrial laser scanners (TLS), also known as ground-based LIDAR, such as the Z+F IMAGER 5016 [41], enable the digitisation of the entire environment in a 360${}^{\circ }$ panorama in the form of a 3D point cloud. During the scanning process, a high frequency rotating mirror deflects the laser beam, and the TLS additionally rotates around its standing axis. This sequential acquisition method produces a high-resolution point cloud of the visible environment. The measurement method is characterised by a very high spatial resolution, but in turn allows only a low temporal resolution.

A profile scanner (TLS in profile mode, 2D) only uses the high-frequency rotating deflection mirror, but there is no rotation around the standing axis (see schematic in Figure 1 on the right side). By reducing the spatial resolution to a single profile, a significantly higher temporal resolution is possible. The spatial resolution within the profile (angular increment) ultimately depends on the combination of the rotation speed of the deflection mirror and the laser measurement rate.

The range measurement of the Z+F IMAGER 5016 works according to the amplitude-modulated continuous wave (AMCW) method. In order to obtain the absolute range value, the phase-shift between the reflected and emitted signal is used, which is induced in an intensity-modulated periodic signal due to its round-trip to the target.

Several wavelengths are modulated onto the carrier wave to resolve phase ambiguities and thus determine an absolute range. In addition, the amplitude (intensity) is provided, which represents the ratio between emitted and received energy.

## 3. Theoretical Comparison of the Sensors for the Contactless Monitoring of Bridges

For application in the field of bridge monitoring, different properties of the two sensors are important. In the following sections, the most relevant of these are compared with each other:

- Section 3.1—Measuring Frequency
- Section 3.2—Measurement Precision
- Section 3.3—Range Resolution
- Section 3.4—Spatial Resolution at the Structure
- Section 3.5—Projection of Displacements

### 3.1. Measuring Frequency

With the IBIS-S, measurements can be carried out with a measuring rate of up to 200 Hz. In general, the maximum usable measuring frequency is related to the range resolution and the maximum range since these two values influence the time required for each measurement sample. However, for the maximum range of 100 m relevant to this study (see Section 1), the maximum possible measuring frequency of 200 Hz can be achieved. A higher measuring frequency, of up to 4.000 Hz, is possible with microwave interferometers from other manufacturers [42].

The usable measuring frequency for the deformation monitoring of bridges with the IMAGER 5016 in profile mode depends on the rotation speed of the deflection mirror which is up to 55 Hz. It should be noted that there is a dependence between temporal and spatial profile resolution: at the same laser measurement rate, a doubled measurement frequency leads to a halving of the spatial resolution. A higher measuring frequency, up to 267 Hz, is possible with the Z+F PROFILER 9020 [43], but then a four times lower spatial resolution must be accepted.

### 3.2. Measurement Precision

For both measuring systems, the precision of the measurement depends significantly on the energy reflected back from the structure and thus on its backscatter properties in the corresponding wavelength band, as the phase measurement accuracy is directly coupled to the signal-to-noise ratio (SNR) of the reflected signal [44].

For the wavelength band of IBIS-S, edges, corners and similar discontinuities in particular act as a good reflector, while in the case of smooth surfaces, such as those of prestressed concrete bridges, little energy is scattered back due to the predominant forward reflection. In contrast, discontinuities are particularly problematic for the profile scanner, due to the mixed-pixel effect [45,46].

For the further comparison of the two measurement systems, however, this must be considered in a more differentiated way, i.e., in connection with the different “illuminated” object surface by each sensor, see Section 3.4.

The manufacturers of both measuring systems give standard deviations for the raw measurements in varying degrees of detail [39,41]. For the IBIS-S, a LOS displacement standard deviation of 0.01–0.1 mm is specified, measured on a stable reference target providing a signal-to-noise ratio (SNR) better than 20 dB. For the IMAGER 5016, as is common practice in the TLS area, the manufacturer provides range measurement standard deviations for different surface reflectivities and ranges. These are based on a fixed laser measurement rate of 127 kHz and range from 0.2 mm to just under 10 mm.

However, those accuracy specifications are not very meaningful in practice and cover only a very small range of applications: The specified reflectivity of the measured structure is usually not known and can also vary spatially. In addition, the measurement geometry in particular plays a decisive role for the specification of realistic measurement uncertainties; it is partly responsible for the occurrence of predominant forward reflection, i.e., the flatter the angle of incidence, the greater the potential for forward reflection and a low SNR. The measurement geometry is, at the same time, crucial for the derivation of the projected vertical deformations. Furthermore, no reflectors are to be used in the application scenario under investigation, so that an accuracy specification based on a defined reflector is not purposeful.

Based on the SNR of the IBIS-S measurement, it is possible to derive an uncertainty measure for the LOS displacement [24]. For TLS in general, the stochastic modelling of the range measurement is possible based on the registered intensities [47,48] and allows a practical determination (insitu) of the range precision. This approach takes into account all effects acting on the measurement process (surface reflectivity, measurement geometry, atmosphere, etc.).

### 3.3. Range Resolution

For the comparison of both measurement systems, the usage of the term range resolution is misleading, because the term corresponds to a different parameter with each sensor.

For the IMAGER 5016, the range is part of the raw measurement. The range resolution is 0.1 mm and is defined by the used size of the modulated fine scale in combination with the implemented phase measurement.

However, with the IBIS-S, the range resolution is independent from the displacement measurement. The range resolution describes the minimum distance required between two objects to distinguish their relative LOS displacement. Since the range resolution is defined in the sensor’s line of sight, the actual resolution at the structure differs. Accordingly, the next section will go into more detail on the spatial resolution at the object for both measuring systems.

### 3.4. Spatial Resolution at the Structure

The range resolution of the IBIS-S depends on the frequency bandwidth authorised by local radio regulation of the transmitted signal. As an example in USA and Europe, the bandwidth is limited to 200 MHz and the range resolution is therefore 0.75 m, see also Section 2.2.

**Figure 2:**
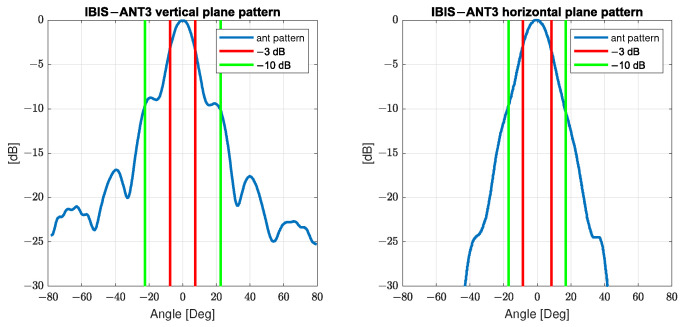
Antenna pattern for the standard antenna of the IBIS-S, vertical and horizontal (based on the User-Manual).

The area at the structure that can be evaluated in this way is limited by the opening angles of the radar lobe, which in turn is defined by the antenna used. For the standard antenna (IBIS-ANT3-H17V15), the manufacturer defines two widths for the main lobe: −3 dB and −10 dB, which additionally differ in vertical and horizontal direction. The parameter quantifies the radar lobe amplitude: −3 dB defines the angular area within the antenna gain of more than 50% of the maximum gain; −10 dB includes the angular area within the antenna gain of more than 10% of the maximum gain, see Figure 2.

The width of the main lobe at −3 dB is defined as 15${}^{\circ }$ vertical and 17${}^{\circ }$ horizontal; for −10 dB it is defined as 45${}^{\circ }$ vertical and 34${}^{\circ }$ horizontal.

**Figure 3:**
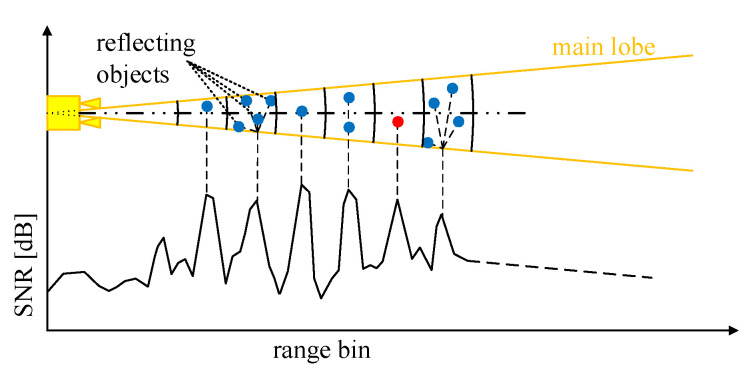
Schematic illustration of the IBIS-S measurement principle in relation to the spatial resolution cells.

Therefore the range resolution of the IBIS-S results in a division of the measuring range into resolution cells: spherical shell segments with an angle range of 15${}^{\circ }$/17${}^{\circ }$ (−3 dB) resp. 45${}^{\circ }$/34${}^{\circ }$ (−10 dB) and a thickness of 0.75 m. In each resolution cell, a sum of the amplitude and phase of all reflections is determined. The contribution of each reflection to the displacement measurement is weighted by its amplitude, which depends on the specific antenna gain and the reflection characteristics. This ultimately leads to the spatial resolution of the line of sight displacements, see schematic in Figure 3 and also Figure 1 on the left side.

The individual resolution cells, defined by the opening angle and bandwidth, are projected onto the structure based on the inclination angle of the sensor, see also Figure 1 and Figure 3. This means that since the choice of the inclination angle (relative to the horizontal) defines the evaluable area at the structure, it also has an influence on the spatial resolution in profile direction: For example, assuming a “smooth” horizontal structure 10 m above the sensor, an IBIS-S inclination angle of 26${}^{\circ }$ results in a spatial resolution between 1 m and 0.76 m along the structure for the area of the −10 dB lobe; if the inclination angle is increased to 44${}^{\circ }$, the achievable spatial resolution lies between 1.4 m and 0.8 m.

Overall, the spatial resolution increases with the horizontal distance to the sensor. Howerver, the larger the inclination angle, the closer the resolution cells are to the sensor, leading to a lower spatial resolution in profile direction for such a configuration and additionally the evaluable area is also decreasing.

Due to the expansion of the radar lobe (mainly horizontal), there is a risk that several reflectors within a resolution cell overlap. Then, the measured displacement in line of sight only represents the (weighted) average behaviour of all reflectors in the resolution cell under consideration. This is particularly problematic if the reflectors differ in their movement behavior due to constructional conditions, e.g., separate directional carriageways or directional tracks on bridges.

In comparison, the actual spatial resolution of a profile scanner is defined by the parameters rotation speed of the deflection mirror, laser measurement rate and divergence angle of the laser beam. The actual spatial resolution is usually lower than the angular resolution specified by the manufacturer due to the following two reasons:

1. Depending on the choice of parameters, the laser spots of successive measurements overlap to a greater or lesser extent, which reduces the actual resolution at the structure surface.
2. The rotation speed induces an additional deformation of the laser footprint (elongation) in profile direction, since a corresponding angular range is always swept during the measurement time. This can be interpreted as a larger “actual” divergence angle or as an increasing overlap of successive measurements according to [46,49].

Another aspect when considering the spatial resolution actually available in practical applications is that the single point precision of a profile scanner is usually not sufficient for the requirements of the application scenario [7]. Therefore, in order to achieve the required precision, averaging is performed using neighbouring measurement points, which, however, further reduces the spatial resolution for profile scanning in favour of a qualitatively better derivation of displacements.

To get an idea of the achievable spatial resolution, two examples are given below:

1. At a measurement frequency of 55 Hz, 20,000 points are measured per profile, which corresponds to a theoretical angular increment of 0.018${}^{\circ }$. If 75 neighbouring measuring points are combined, the actual available angular increment is reduced to 1.35${}^{\circ }$, which corresponds to a spatial resolution of 0.24 m at 10 m distance.
2. If the measuring frequency is reduced to ≈14 Hz, a spatial resolution of 0.24 m at a distance of 40 m can still be achieved.

It can be seen that statements about the spatial resolution can only be made in connection with the selected measurement frequency and the existing measurement setup. In addition, special features of the object surface (ideally planes are used) may have to be taken into account. Compared to the IBIS-S, however, the spatial resolution can be freely adjusted within certain limits.

The spatial resolution perpendicular to the profile direction is defined for both systems by the opening angle of the measuring beam. For the IBIS-S this is 17${}^{\circ }$/34${}^{\circ }$ (−3 dB/−10 dB, horizontal) for the IMAGER 5016, it is approx. 0.6 mrad, which corresponds to a factor of approx. 500/1000 between the sensors. At a distance of 10 m this means a footprint width of ≈3 m/6 m for the IBIS-S and 0.006 m for the IMAGER 5016.

### 3.5. Projection of Displacements

The purpose of using these sensors for the deformation monitoring of bridges is to obtain deformations in a defined direction (mostly vertical or horizontal). Therefore, the “raw” measurements must be projected in the required direction. For the projection, it is usually assumed that the vertical displacement of a bridge is predominant, while possible horizontal components are considered negligible. In the first part of this section we will follow this assumption before discussing the effects of multiple displacement components.

#### 3.5.1. Single Displacement Component

Since the raw measurements of a profile scanner consist of distance and internal angle measurements, the projection of the deformations is inherent and possible with high accuracy. The manufacturer specifies an angle accuracy of 0.004${}^{\circ }$ for the IMAGER 5016. The horizon reference is realised via an internal dynamic compensator, which operates in the same accuracy range as the angle encoders. In addition, the dynamic compensator makes it possible to detect low frequency movements of the sensor during the measurement and to correct them if necessary.

The IBIS-S has no (additional) internal sensors to determine projection angles. Therefore, external sensors are required to derive the corresponding value for each resolution cell. The projection angle for a resolution cell results from the inclination angle of the radar head and the vertical position within the radar lobe: at 44° radar head inclination in combination with an opening angle of the radar lobe (−10 dB) of ± 22.5°, projection angles between 21.5° and 66.5° can thus occur. At 26° radar head inclination the projection angles vary between 3.5° and 48.5°; thus the same projection angle can occur despite different inclination angles of the radar head. In order not to reduce the accuracy of the measurement by the projection, the recording geometry must be well known.

If the situation is reduced to a 2D case, this consists of a right-angled triangle that is spanned by the zero point of the IBIS-S and the theoretical “reflector” on the structure. Accordingly, two elements must be known in order to determine the respective projection angle. With a “perfectly smooth” horizontal (2D) structure as shown in Figure 1, the projection angle for each resolution cell could be determined, for example, on the basis of mean LOS ranges in combination with the vertical distance of the IBIS-S zero point to the underside of the bridge. The projected displacement *v* can be calculated from the LOS displacement $\Delta r_{LOS}$ using the range *R* and the height *h*:

(2) $$v=\frac{\Delta r_{LOS}\cdot R}{h}$$

In order to state the uncertainty of a projected displacement according to (2), geometric uncertainties of the measurement configuration have to be discussed in addition to the influence of the uncertainty of the LOS measurement: Depending on the structure of the underside of the bridge, different heights and LOS ranges result for the individual resolution cells, depending on the position of the virtual “main reflector”. For the 2D case the possible variation of the “reflector” in range and height is introduced as the uncertainties $s_{R}$ and $s_{h}$. In combination with the uncertainty for the LOS displacement $\Delta r_{LOS}$ the standard deviation of the projected displacement $s_{v}$ can be derived:

(3) $$s_{v}^{2}={\left(\frac{R}{h}\right)}^{2}\cdot s_{\Delta r_{LOS}}^{2}+{\left(\frac{\Delta r_{LOS}}{h}\right)}^{2}\cdot s_{R}^{2}+{\left(\frac{\Delta r_{LOS}\cdot R}{h^{2}}\right)}^{2}\cdot s_{h}^{2}$$

Without additional information about the position of the virtual “reflector” in the resolution cell, the possible height variation depends entirely on the measured structure and the possible LOS range variation is limited only due to the size of the resolution cell.

**Figure 4:**
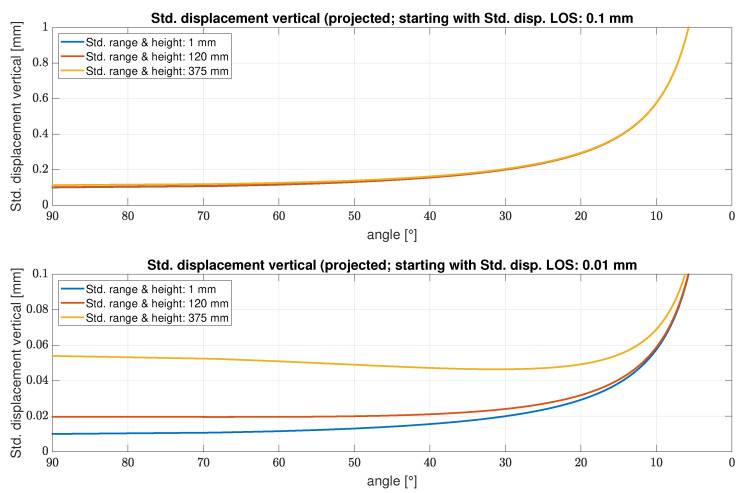
Uncertainty of the projected displacements (vertical) for different standard deviations of the LOS displacement (upper vs. lower graphic), projection angles and precision of range and height for the resolution cells.

In order to estimate the influences of the discussed uncertainties on the standard deviation of the projected displacement in general, angle-dependent measurement configurations are considered under three different uncertainty assumptions for the geometric quantities *r* and *h*, respectively. The lower and upper bounds of the LOS measurement accuracy (manufacturer specifications) each generate an investigation scenario, see Figure 4 top and bottom.

The upper graph shows that with a standard deviation for the LOS displacement of 0.1 mm, the standard deviation of the projected displacement increases with decreasing angle, with a much greater deterioration below 30${}^{\circ }$. However, the precision of range and height do not play a significant role in this configuration, even with a precision of only 375 mm (half the size of the range resolution) there is hardly any difference.

Assuming a much better precision for the LOS displacements of 0.01 mm (see lower graph), the picture is similar, the standard deviation of the projected vertical deformations decreases considerably, especially in the low angular range. However, in contrast to the first scenario, the uncertainties of distance and height also have a significant influence on the precision of the projected displacement.

This very simple 2D approach outlines only a small part of the problem. In practice, the situation is usually much more complicated and must be discussed in corresponding detail (see Section 4). Especially with complex structures of the bridge underside and larger distances to the sensor (increasing extension of the radar lobe), the danger that several “main reflectors” are located within one resolution cell increases, which makes a correct derivation of a projection angle almost improbable. If, moreover, a different motion behavior of the reflectors is present, a mixed signal is generated which, in the best case, can be recognized and sorted out as such.

#### 3.5.2. Multiple Displacement Components

Up to this point, it has been assumed that only a vertical displacement of the bridge structure exists due to an applied load. However, if an additional significant horizontal displacement is present, the situation is becoming more complex.

Such horizontal displacements in the longitudinal direction of the bridge can already be caused by the vertical deflection of the bridge, since free bearings in particular move horizontally in the direction of the center of the span. Acceleration when the train enters the bridge or during braking can also cause vertical deflection. Horizontal displacements transverse to the bridge can additionally occur if for example the bridge lies in a curve (sinusoidal run). The maximum values of both horizontal displacement components are usually in a very low millimetre range. In principle, it can be assumed that the vertical displacement is dominant (by far).

For the derivation of vertical displacements with the profile scanner, such small horizontal displacements are negligible; these deviations are not registered at all. However, since the profile scanner is a 2D measurement sensor, these can be measured directly at suitable locations (vertical areas) if the displacement is in profile direction.

In contrast to this, however, the IBIS-S is a 1D measuring system, thus in an inclined measurement setup, the components cannot be separated by a single LOS measurement which is comprised of more than one displacement component, leading to a deviation in the projected vertical displacement. If the IBIS-S measures vertically upwards (without projection) then a small horizontal displacement is also irrelevant.

Alternatively, multiple displacement components can be separated by using more than one IBIS-S, as described by [50,51] or an alternative sensor for the derivation of horizontal displacements. The transformation of the measurements $\Delta r_{LOS}$ to a three-dimensional displacement vector $\Delta r_{p}$ is derived by the geometric relation between the sensors and the targets. With the known target coordinates $P_{T}$ and sensor coordinates $P_{i}$, the distances $d_{i}$ are defined:

(4) $$\begin{array}{c} d_{i}={∥P_{T}-P_{i}∥}_{2} \end{array}$$

The partial derivatives of the distances with respect to the target coordinates result in the Jacobian matrix *J*, defining the transformation as

(5) $$\begin{array}{c} \Delta r_{LOS}=J\cdot \Delta r_{p} \end{array}$$

By inverting the Jacobian matrix, the three-dimensional displacement vector results:

(6) $$\begin{array}{c} \Delta r_{p}=J^{-1}\cdot \Delta r_{LOS} \end{array}$$

Usually, only two displacement components are relevant for the most common measurement setups of IBIS-S. The Jacobian matrix is then modified to exclude the corresponding line and column of the third component, enabling the transformation with only two sensors. Similar to the uncertainty analysis of the single displacement component, the measurement uncertainty can be propagated in the case of multiple components as well. The measurement uncertainty $s_{\Delta r_{LOS}}$ is propagated with the Jacobian matrix:

(7) $$\begin{array}{cc} \Sigma_{\Delta r_{LOS}} & =s_{\Delta r_{LOS}}\cdot I \end{array}$$

(8) $$\begin{array}{cc} \Sigma_{\Delta r_{p}} & =J^{-1}\cdot \Sigma_{\Delta r_{LOS}}\cdot {J^{-1}}^{T} \end{array}$$

As discussed earlier, the uncertainty of the geometric relation has an additional influence on the uncertainty of the projected displacements and could be modelled with a Monte-Carlo simulation.

## 4. Contactless Monitoring at a Railway Bridge

**Figure 5:**
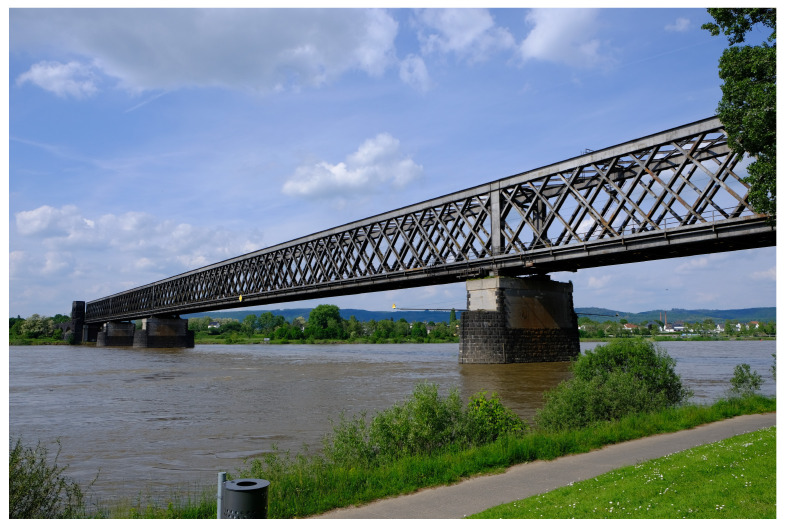
Railway framework bridge under investigation.

In the previous section, problems were pointed out in particular for the IBIS-S. In order to illustrate these problems, which up to now have only been discussed theoretically, investigations on a large railway framework bridge are shown in the following section. The investigated structure is a double-track steel bridge consisting of a four-span continuous framework girder, see Figure 5. The goal of the investigation was to derive spatially distributed deformations at the underside of a bridge span on the south side with a span width of approx. 82 m.

Since the arising problems are to be understood as a synthesis of IBIS-S measurement configuration (acquisition geometry) and the complex-structured measurement object, we first take a closer look at the bridge structure in the relevant area. For this purpose, 3D laser scans of the IBIS-S positions and the relevant bridge section were taken with a TLS, which enables the visualisation of the IBIS-S spatial resolution on the scanned bridge.

**Figure 6:**
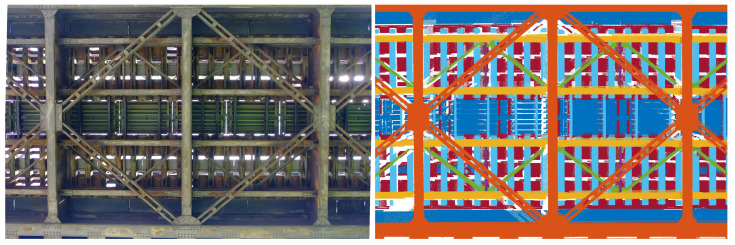
Complex framework of the underside of the bridge: Photograph vs. color-coded (height) laser scan.

Figure 6 shows a section of approx. 12 m of the underside of the bridge (3 cross girders) as a photograph and as a 3D laser scan, with the individual components highlighted in different colours using the z-coordinate. This section is representative for the entire underside of the bridge. The bridge consists in addition to the two large main longitudinal girders, which are connected by cross girders (orange), of four smaller longitudinal girders (yellow), on which the actual tracks rest (light blue). Wind bracing runs between the large longitudinal girders (orange) and between each of the smaller pairs of longitudinal girders (yellow) there is also an additional slinger bracing (green).

**Figure 7:**
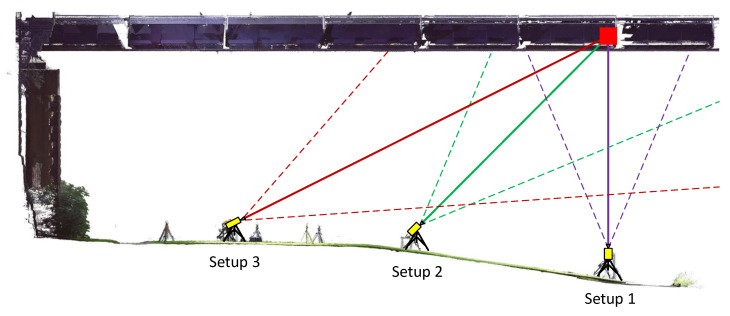
Side view of the bridge (laser scan) showing the schematic recording geometries of the 3 setups.

The application scenario defined in Section 1 requires a spatial resolution in order to enable an efficient monitoring application of the sensor. With the IBIS-S, however, this is only the case if the sensor is inclined, which means that the evaluable range and the spatial resolution at the object depends on the distance to the object and also the sensor inclination angle (relative to the horizontal). The inclination of the IBIS-S increases the measurable area on the object: An inclination angle of 44° results in a measurable area of 20 m in a height 10 m above the sensor, whereas an inclination angle of 26° results in a measurable area of 138 m. Both values are given for the angle range of the −10 dB lobe. To obtain qualitatively better measurements, it is advisable to use only the −3 dB range, but then the measurable range is significantly smaller. If the negative effects discussed in the previous section are ignored for the time being, it would make sense for the user to work with the largest possible inclination angle in order to obtain the largest possible evaluable area. To take this (only theoretically sensible) approach into account, a total of three positions (setup 1 to 3) were realised with the IBIS-S, see Figure 7.

Figure 7 shows the corresponding acquisition geometries in a side view of the 3D scan. The used inclination angles (relative to the horizontal) of 90°, 44.4° and 26.4° as well as the opening angles of the radar lobe (−10 dB, dashed) are shown. For all three configurations, the same connection to the (sixth) cross beam of the bridge span was targeted. It should be noted that only setup 2 and 3 (inclined) offer a spatial resolution, setup 1 (vertical) therefore does not correspond to the defined application scenario and was additionally implemented to verify the comparability of the measurements of both sensors.

**Figure 8:**
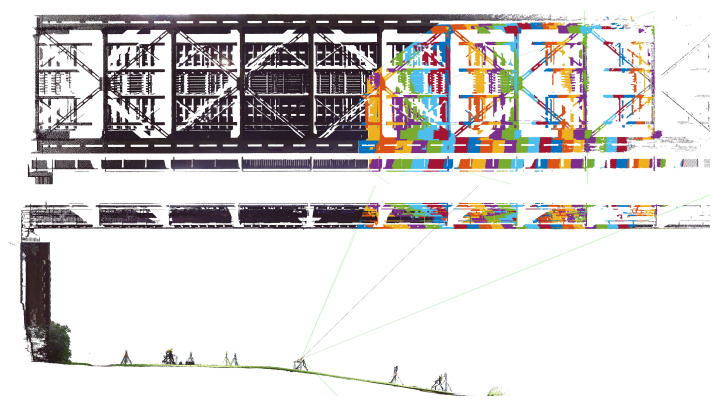
Top and side view of the bridge with the projected resolution cells (color-coded) of the IBIS-S for Setup 2.

Based on the measurement configuration of Setup 2, the following description explains how the “view of the IBIS-S” on the object is derived from the 3D-scan: First, the used inclination angle of the IBIS-S is calculated based on a plane modelling of all scanned casing sides. With the inclination angle and the defined zero point of the IBIS-S (back of the casing in the middle between the two connectors), the resolution cells can then be projected onto the object, see Figure 8 for Setup 2.

With this visualisation, it is possible to identify potential reflectors of the microwave interferometer signal within individual resolution cells in order to derive the optimal geometrical parameters for determining the projected displacement (projection angle). On the other hand, Figure 8 illustrates the problems already discussed at the end of Section 3 regarding the correct localisation of reflectors and the size of the aperture angle of the radar lobe, which can easily prove insurmountable with complex object structures:

- Due to the open framework structure of the bridge, a large number of potential “reflectors” and thus good reflection properties (high SNR values) can be expected. However, in the side view (Figure 8 lower graphic) it can be seen that an area of approx. 1.8 m vertically can lie within a resolution cell, which makes the identification of the “correct” reflector almost impossible and thus leads to a large uncertainty in the determination of the projection angle.
- The size of the aperture angle is also problematic for the differentiated consideration of details at complex object structures. In Figure 8 upper graphic it can be seen that already in the first (visualised) resolution cells more than half of the bridge width is contained in the −10 dB radar lobe. At the end of the evaluable range (right part of Figure 8 upper graphic), the radar lobe already covers almost the entire width of the bridge, so that (always) both directional tracks are detected in one measurement. It must therefore be assumed that in such resolution cells a mixed signal is generated from reflections on the loaded and unloaded track.

In the following section, the results of this study will be presented, especially with regard to the latter problem. However, prior to this, the comparability of the derived vertical deformation of the two sensors is shown on the basis of setup 1 (vertical).

### 4.1. Setup 1 (Vertical): Basic Comparability

**Figure 9:**
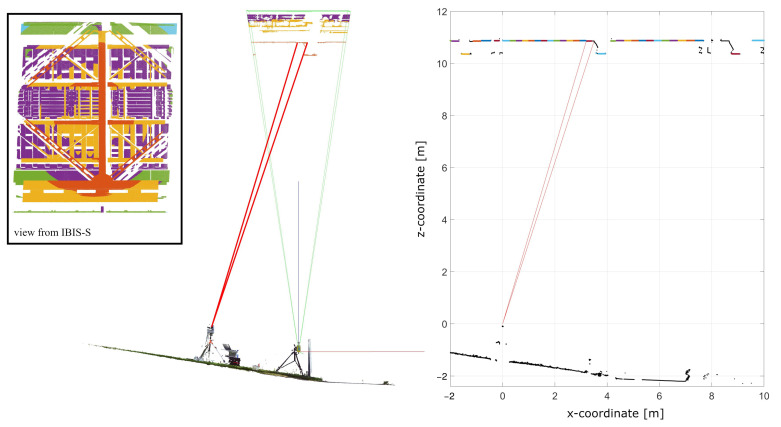
Measurement configuration of IBIS-S and IMAGER 5016 in profile mode for setup 1. On the left side a 3D scan of both sensors is shown, including the color coded (range bins) view of the bridge underside from the IBIS-S. On the right side a cutout of a processed profile of the IMAGER 5016 is shown, which is color coded based on the spatio-temporal processing scheme from [7].

In order to demonstrate the basic comparability of the two sensors, they were set up side by side, see Figure 9. The IBIS-S pointed vertically upward (angle of inclination 90°, see also Figure 7 Setup 1), so no projection is necessary, but there is also no usable spatial resolution at the object. The resolution cells lie one behind the other and due to the open framework structure several resolution cells intersect the structure, see Figure 9 top left side (view from IBIS-S).

The vertical distance from the IBIS-S to the connection of the small longitudinal girder to the cross girder is 11.8 m. Accordingly, resolution cell 16 is relevant for the further comparison; it mainly contains the cross girder and the small longitudinal girder and is shown in Figure 9 on the left side in red. The other resolution cells contain one main longitudinal girder, the lateral bracing, the railway sleepers, metal plates, etc. (shown in yellow, purple and green).

Depending on the resolution cell, the −10 dB lobe can cover the entire bridge width and a section of up to 8 m in the longitudinal direction of the bridge, see Figure 9. The −3 dB lobe, which dominates in this case, covers only the targeted longitudinal girder, in particular for resolution cell 16 (red), as well as a section of the cross girder.

With the IMAGER 5016, a profile was measured along the corresponding longitudinal beam, see Figure 9 on the right side. Based on the data driven spatio-temporal processing scheme [7], the relevant measurement information was extracted automatically and separated in corresponding classes (see different colors in the profile in Figure 9 on the right side). From this processed data set, an approx. 25 cm long section directly at the connection to the cross girder was chosen, which contains 73 points per profile. The boundaries of the selected area are highlighted with red lines.

**Figure 10:**
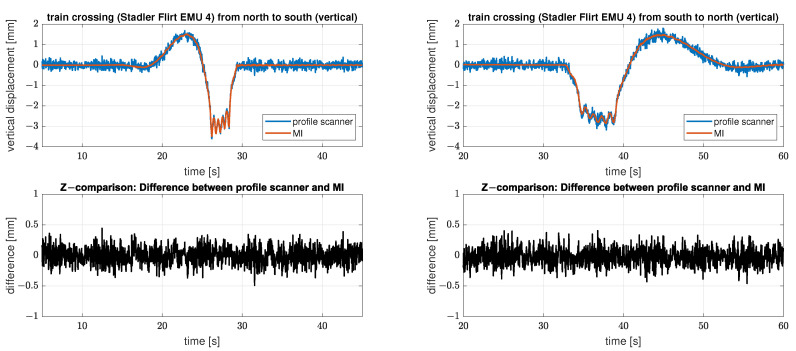
Comparison of the derived displacements with both measuring systems (profile scanner and MI) at cross girder 6. Line 1 shows the measurements of the IMAGER 5016 in profile mode in blue and the IBIS-S measurements in red (no projection necessary) and line 2 displays the respective differences in black.

Based on those datasets Figure 10 shows two crossings of a Stadler Flirt EMU 4 with a duration of 40 s each. The crossings differ in train speed and direction of travel. Due to the construction of the bridge as a continuous framework girder, the bridge span is “lifted” as soon as the train is on the adjacent bridge span. The characteristic of a train crossing therefore always consists of at least one deformation in the negative and positive range, the order depends on the direction of travel of the train.

Figure 10 shows the results of the IMAGER 5016 in profile mode in blue and of the IBIS-S measurements in red. The deviations from each other are in the range of a few tenths of a millimetre with a maximum negative deformation of just under 4 mm.

These two representative results show the comparability of the two sensors, as there are only deviations within the measurement uncertainty of the respective sensor and no visible systematic deviations.

### 4.2. Setup 2 and 3 (Inclined): Spatial Resolution

In contrast to setup 1, the IBIS-S was inclined in setup 2 and 3 to allow the derivation of spatially distributed displacements. The position and the inclination angle of the IBIS-S were changed for each setup.

For Setup 2 the IBIS-S was located under the fourth cross girder and inclined at an angle of just under 45°, still targeting a connection to the 6th cross girder of the bridge span, see Figure 7 and Figure 8. A total of 29 resolution cells intersect the bridge structure and cover a horizontal area of just under 25 m. The LOS distance to the targeted connection at the 6th cross girder is 14.72 m in this case.

**Figure 11:**
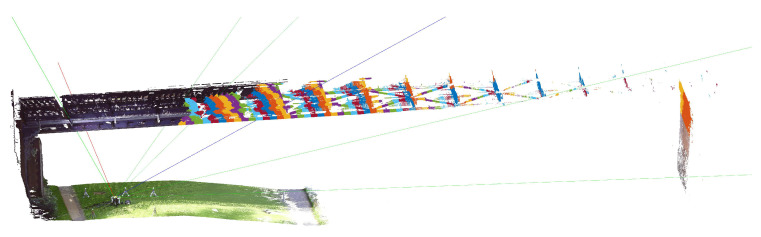
Perspective view of Setup 3 with color-coded resolution cells for the IBIS-S.

To obtain a larger evaluable area (spatial resolution) at the bridge the IBIS-S was inclined even more, with an angle of approx. 26° in setup 3, still targeting a connection to the sixth cross girder of the bridge span, see Figure 11 and also Figure 7. The sensor was positioned under the second cross girder, resulting in over 70 resolution cells intersecting the bridge structure and covering a horizontal area of over 60 m. In the direction of the bridge center, the measuring range was limited by the first bridge pillar. The LOS distance to the targeted connection at the sixth cross girder is 22.67 m in this case.

**Figure 12:**
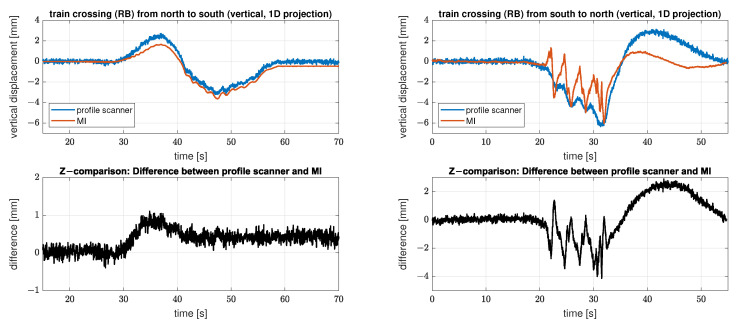
Comparison of the derived displacements with both measuring systems (profile scanner and MI) at cross girder 6 for setup 2 (left side) and setup 3 (right side). Line 1 shows the measurements of the IMAGER 5016 in profile mode in blue and the 1D-projected IBIS-S measurements in red, and line 2 displays the respective differences in black. Note the different scaling of the differences between the two train crossings.

In contrast to Figure 10, the time series of the registered deformations of the two measurement systems now do not match properly for either Setup 2 or 3, see Figure 12. The figure shows on the left side the results of setup 2 at cross girder six and on the right side the results of setup 3 also at cross girder six.

The time series of the analysed bridge part on the left side (setup 2) is “lifted” as soon as the train is on the adjacent bridge span and especially in this part, the two sensors record an increasing difference in the registered deformations (difference of up to 1 mm). After the measurements pass the “zero line” (approx. second 40) there is an offset between the two sensors of approx. 0.5 mm, which is constant afterwards. While the measurements of the IMAGER 5016 in profile mode (blue) return to the initial level, the offset remains for the IBIS-S measurements (red).

In the following it can be shown that the difference between the results of both measurement systems is caused by an additional horizontal displacement component of the observed bridge span. However, since a single LOS measurement of the IBIS-S cannot resolve separate information about two different displacement directions, both components are projected in the vertical axis; therefore, the displacement realised via the projection deviates from the true vertical displacement.

The time series of the IBIS-S on the right-hand side of Figure 12 (Setup 3) shows another, much more drastic effect. In addition to the deviations in the positive displacement range (latter part of the time series) there are large fluctuations in the negative displacement range (load phase). These deviations are most likely caused by the presence of multiple reflectors with different deformation behaviour in the particular resolution cell combined with the horizontal displacement of the bridge field. The advantage with this time series is that, in contrast to the left side, it can be recognised directly that the measurement of the IBIS-S for this resolution cell is strongly distorted and therefore not usable for further analysis.

Unlike with the IBIS-S, the horizontal displacement component has no significant influence on the measurements of the IMAGER 5016 in profile mode, both time series are smooth and follow the same (realistic) pattern as before. Furthermore, it is even possible to record the horizontal displacement at suitable locations in the measured profile [18].

Due to the acquisition geometry, there are no suitable locations to determine horizontal displacements at cross girder six. Therefore the following investigations related to the impact of horizontal displacements are performed at cross girder four and five. Both cross girders are particularly suitable for illustrating the effects of a horizontal displacement, as the measurement geometry is well suited for both determination by means of profile scanning and also for microwave interferometry.

**Figure 13:**
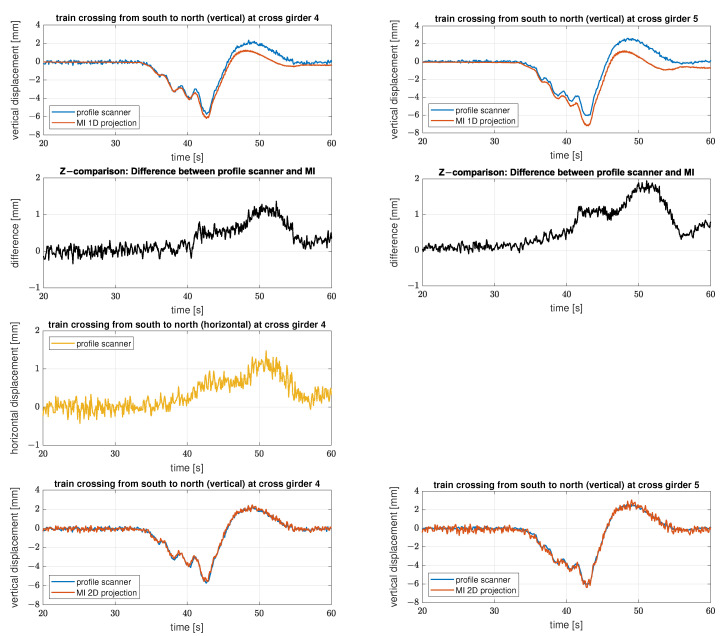
Comparison of the derived displacement with both measuring systems (profile scanner and MI) at cross girder 4 (left side) and cross girder 5 (right side) for setup 3. The figures in line 1 show the measurements of the IMAGER 5016 in profile mode in blue and the 1D-projected IBIS-S measurements in red, and in line 2 the respective differences are displayed in black. In line 3 the horizontal displacements measured with the IMAGER 5016 in profile mode at cross girder 4 are presented in yellow. Line 4 shows the measurements of the IMAGER 5016 in profile mode in blue and the 2D-projected IBIS-S measurements in red.

Figure 13 displays the results of the measurements of the IMAGER 5016 in profile mode and of the IBIS-S for cross girder four on the left side and cross girder five on the right side. For the profile scanner, four consecutive profiles were combined to increase the spatial resolution in the evaluation area. This is particularly useful for determining the horizontal displacement, as the measurable sections are relatively small. Since the train crossings do not contain any high-frequency components, the resulting low sampling rate is quite sufficient for the analysis in the time domain.

In the first line the vertical displacements for the measurements of the IMAGER 5016 in profile mode in blue and the IBIS-S measurements using the 1D-projection in red (see Section 3.5.1 and compare with Figure 12) are shown.

The second line depicts the differences between those two time series in black. The differences follow the same pattern as shown in Figure 12 on the left side. The comparison of the differences from cross girder four and five show that the flatter the projection angle the bigger the effect due to the horizontal displacement. In the case of cross girder four, the projection angle is approx 45°, so the horizontal displacement is translated 1:1 (erroneously) into the deviations from the nominal deformation (compare line 2 and 3). In the case of cross girder five, the projection angle is smaller, so that the horizontal displacements have an even stronger influence on the (incorrectly) projected vertical displacements.

The horizontal displacements measured with the profile scanner are presented in line 3 in yellow. The displacement could only be measured at cross girder four, therefore only one time series is shown. Due to the projection angle of approx. 45°, the shape and scale of the horizontal displacements correspond directly to the vertical deviations between the two measuring systems evaluated at cross girder four.

The last line depicts the results of the measurements of the IMAGER 5016 in profile mode and of the IBIS-S using the 2D projection (see Section 3.5.2). By combining the IBIS-S measurements with the horizontal displacements determined by the IMAGER 5016 in profile mode, the previously existing deviations are completely corrected; therefore we can assume that the horizontal displacements at cross girder four are representative for both cross girders. It is thus obvious that all systematic effects are compensated by the 2D projection and that only a slightly increased noise level remains for the IBIS-S measurements, due to the SNR of the horizontal displacement measurements with the IMAGER 5016 in profile mode.

The roughening effect that can be seen in the corrected IBIS-S measurement series depends not only on the SNR of the horizontal displacements but also on the projection angle. According to variance propagation, noisier IBIS-S measurement series (corrected) are observed at lower projection angles (cf. left and right columns).

The observed effect can also be proven mathematically. Equation (8) enables the estimation of the resulting uncertainty. The estimation considers the geometric relations as well as the measurement uncertainties for the respective sensors. The horizontal time series measured with the IMAGER 5016 in profile mode has an uncertainty of 0.15 mm, while the IBIS-S achieves the best possible uncertainty of 0.02 mm due to the high SNR at the crossbeams four and five. After the 2D projection, the uncertainty of the resulting vertical displacements is estimated to be 0.16 mm at cross girder four and 0.25 mm at cross girder five. Which also fits very well to the visual impression.

## 5. Conclusions

This paper compares two contactless measurement systems, microwave interferometry and profile scanning, both on a conceptual level and in the context of a joint practical application in monitoring a bridge structure. It was demonstrated that both measurement systems can in principle be used to solve dynamic monitoring tasks. In particular, their high spatial resolution enables an extremely efficient use, since a large number of object points can be detected simultaneously.

However, this basic suitability is only possible under certain restrictions, especially in the case of the microwave interferometer. In principle, a single IBIS-S can only be used appropriately if the deformations to be detected occur exclusively in a vertical or a horizontal direction. However, even under this condition, the following problems must still be taken into account.

When measuring with an inclined radar head, errors in the projection of the LOS displacement measurements can hardly be avoided. This does not apply to the special case when measurements are made directly in the direction of the deformation, which has been excluded from these considerations, since in such a configuration no spatial resolution can be realized at the structure.

- Even if initially the precise detection of LOS displacements at discrete points of the structure by the IBIS-S is assumed, the correct inclination determination of the LOS direction can rarely be realized with the precision that would be necessary to maintain the low uncertainty of the measured LOS displacement during the projection process onto the vertical or horizontal deformation component.
- However, for an error-free determination of the projection angle, not only the precise inclination determination of the LOS direction is crucial, but also the correct identification of the reflector on the structure to which the determined LOS displacement is assigned. In Section 4, this problem was discussed in detail and it was shown that a precise identification at complex structures with multiple potential reflectors in every resolution cell is almost impossible. This in turn leads to uncertainties for the projected deformations that are many times larger than the uncertainty of the determined LOS displacement.

In addition to the correct localization of a single reflector, the existence of several reflectors with different deformation behaviour in a resolution cell is therefore a separate problem. This can lead to deviations of up to 66% (4 mm) relative to the maximum deformation (6 mm) for an affected resolution cell, as shown in the example in Section 4.2. Because such a time series usually has strong distortions compared to the time series of other resolution cells, it can be relatively easily identified as erroneous and eliminated, which of course reduces the spatial resolution of the deformation accordingly.

Furthermore, it must be assumed in general that occurring deformations consist of both vertical and horizontal components, which is, however, often neglected in practice. The consequences that arise when 2D deformations are unknowingly recorded with a 1D measurement system are discussed in Section 4.2. Therein, 2D displacements acquired with the profile scanner are contrasted with the 1D displacements of the microwave interferometer. Since the LOS measurements of the microwave interferometer do not contain any information on how to correctly decompose them into horizontal and vertical displacement components, a one-sided projection onto the vertical displacement component creates a systematic distortion in all time series (see Figure 13). In addition, we show how to correct the distorted IBIS-S time series using the IMAGER 5016 in profile mode at suitable locations.

Especially measurement series which are not so clearly distorted (e.g., compared to time series with multiple reflectors) are a potential danger. In the basic shape, no outstanding distortions are visible at first, the distortion only becomes apparent when comparing the measurements with those of another sensor. Nevertheless, even after the “wrong” projection, the low LOS measurement uncertainty results in a very smooth curve, which is considered synonymous with high precision. In the end a much lower uncertainty is assumed, which, however, cannot be achieved due to incalculable systematic effects, and this error will not be detected in standalone use.

In contrast, the problems mentioned above do not occur with the profile scanner as a 2D measurement system, since the projection is inherent due to the internal angle encoder. Furthermore, it is even possible to record the horizontal and vertical deformation simultaneously and use these measurements for correction and subsequent validation of the distorted IBIS-S measurements. For these reasons, profile scanning is more suitable for standalone use in dynamic monitoring applications. While the microwave interferometer offers a higher measurement frequency and a lower noise level in LOS, the successful application depends too much on limiting surrounding conditions and prior knowledge of the structural response. In addition, deformations can only be derived in large resolution cells, and a detailed analysis of the structure is therefore not possible.

## Data Availability

The data presented in this study are available upon request from the corresponding author.
